# Severe Crohn’s Disease With Intra-abdominal Fistula: First Reported Case From Tanzania

**DOI:** 10.7759/cureus.21277

**Published:** 2022-01-15

**Authors:** Nadeem Kassam, Omar Aziz, Eric Aghan, Melchisedeck Mandwa, Caroline Ngimba, Hanifa Mbithe, Salim Surani, Casmir Wambura

**Affiliations:** 1 Internal Medicine, Aga Khan University Medical College, East Africa, Dar es Salaam, TZA; 2 Medicine, Aga Khan University Medical College, East Africa, Dar es Salaam, TZA; 3 Medicine, Aga Khan Hospital, Dar es Salaam, TZA; 4 Family Medicine, Aga Khan Hospital, Dar Es Salaam, TZA; 5 Internal Medicine, Aga Khan University Medical College, East Africa, Dar-es-Salaam, TZA; 6 Internal Medicine, Aga Khan Hospital, Dar Es Salaam, TZA; 7 Anesthesiology, Mayo Clinic, Rochester, USA; 8 Medicine, Texas A&M University, College Station, USA; 9 Gastroenterology, Aga Khan University Hospital, Dar Es Salaam, TZA

**Keywords:** crohn’s disease (cd), diarrhea, gi fistula formation, abdominal pain, enterocolitis, irritable bowel syndrome

## Abstract

We describe a case of Crohn's disease occurring in a young Tanzanian female. Crohn’s disease is rare in Africa and not encountered normally. The presentation of Crohn’s disease overlaps with many other abdominal disorders that are common in an African setting, such as tuberculosis and schistosomiasis. The disease is probably underdiagnosed in Africa due to limitations in diagnostic testing and rarity.

## Introduction

Crohn’s disease (CD) is a chronic relapsing inflammatory bowel disease (IBD). It is chiefly characterized by a transmural granulomatous inflammation affecting any part of the gastrointestinal tract, most frequently the terminal ileum [1]. CD arises from a complex interaction between genetic and environmental factors [2]. The incidence and prevalence of CD appear to be growing globally but vary by geographic location and are considered uncommon in an African setting. The prevalence of tuberculous enterocolitis and schistosomiasis in Africa is considered an impediment to effectively diagnosing CD since both have clinical and histological similarities [3-5]. The distinction of CD from tuberculosis is relatively even more challenging if the lesion is granulomatous [5]. A case of histopathologically confirmed CD is presented here because of the paucity and clinical curiosity of this entity.

## Case presentation

A 24-year-old female of African origin was admitted to our hospital with a six-month history of fresh persistent bloody diarrhea and joint pain. She reported that the diarrhea was foul-smelling, mucoid, and infrequently watery. Her bowel frequency was progressively increasing over time to around 8 to 10 bowel movements per day associated with crampy abdominal pain especially on defecation. Her symptoms were associated with unintentional weight loss of around 10 kg over the past three months. The joint pains were localized to both the ankles and the metacarpophalangeal (MCP) joints, which were worse in the morning. She reported no fevers throughout her illness but developed a few episodes of non-bilious vomiting one day prior to the admission. The patient was a primary school teacher with no history of alcohol consumption or cigarette smoking and denied use of any chronic medication. She had multiple outpatient visits to several facilities for which she was treated for infectious diarrhea with no improvement. She tested negative for HIV, was given steroids for her joint pain, and was asked to adjust her diet. Her past medical history was unremarkable for chronic disease or previous surgeries; she was nulliparous and had normal menstrual cycles.

**Figure 1:**
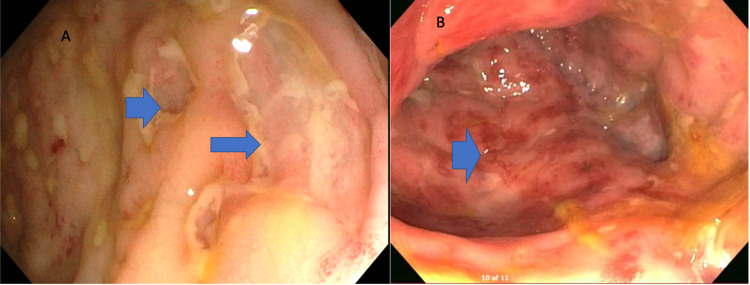
(A) Mucosa of the colon revealing presence of skip lesions and linear ulcerative lesions. (B) Erythema in the gastric mucosa and cobblestone appearance.

On examination, the patient was ill-looking, afebrile, mildly pale, and dehydrated with three pustular lesions noted on her face. At the time of admission, the patient had normal vitals, apart from mild tachycardia (heart rate of 110 beats/minute). Abdominal examination revealed mild epigastric tenderness, and a digital rectal examination was normal. On local examination of the perineum, she had multiple excoriations and three well-demarcated shallow ulcers with regular margins having a diameter of less than 1 cm. A musculoskeletal examination revealed evident swelling of both her ankle joints and MCPs. There was no back deformity noted but was tender on both sacroiliac joints. The patient’s initial investigation revealed the following: hemoglobin (Hb) of 9 g/dL (normal: 12.3-15.2 g/dL), an elevated erythrocyte sedimentation rate (ESR) at 73 mm/hour (normal: 0-20 mm/hour), and mildly decreased albumin level of 31.05 g/L (normal: 34-54 g/L). The patient underwent esophagogastroduodenoscopy and colonoscopy, which revealed severe pancolitis, as shown in Figure 1.

**Figure 2:**
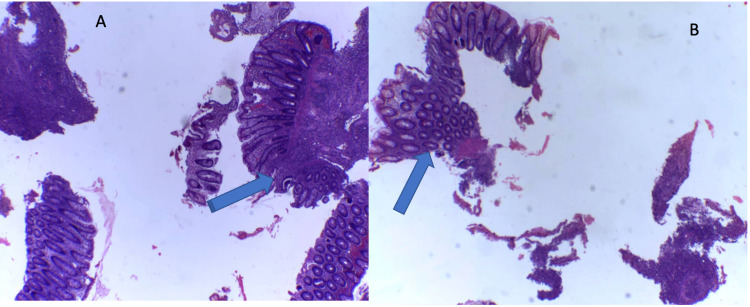
(A, B) Hematoxylin and eosin staining (x10) showing a section of the colon with transmural inflammation with granulomatous inflammation in the submucosa.

**Figure 3:**
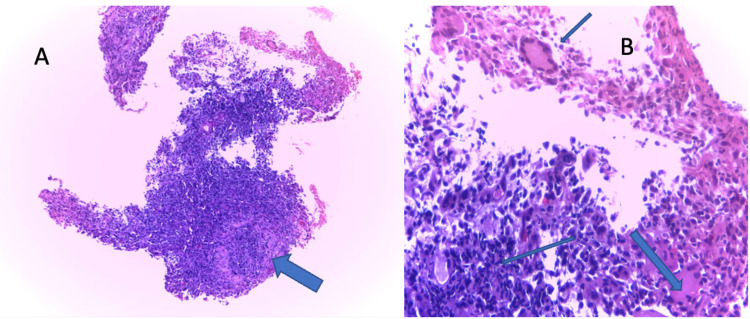
(A) Hematoxylin and eosin staining (x20) of the above section of the colon showing a non-caseating granuloma. (B) Hematoxylin and eosin staining (x40) showing epithelioid cells, multinucleated giant cells, and chronic inflammatory cell infiltrates

A biopsy of the colon was taken, histology of which revealed transmural inflammation with granulomatous inflammation suggestive of CD (Figure 2). Esophageal and gastric tissue histology showed features suggestive of active chronic gastritis (Figure 3). The patient was diagnosed with severe CD with IBD associated with arthritis.

**Figure 4:**
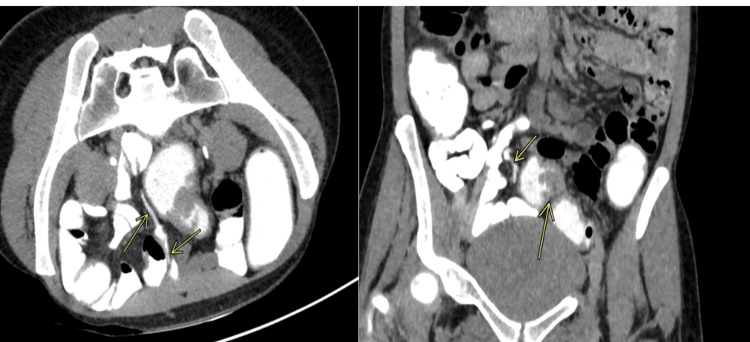
(A, B) Axial and sagittal CT images showing fistula in the abdomen.

The patient was started on sulfasalazine 500 mg every 12 hours and prednisolone 40 mg daily. On return to the clinic, she reported persistent but mildly improved symptoms. Abdominal computed tomography (CT) was performed, as seen in Figure 4, which revealed bowel-wall thickening, strictures at the distal ileum and rectosigmoid junction, and ileocolic and ileoileal fistula.

## Discussion

CD causes inflammation of the digestive tract, mostly affecting the ileum [1]. CD is considered a disease with bimodal distribution having two peaks of onset, the first peak between age 20 and 30 years, as seen in our patient, and the second peak at age 50 years with a median age of 30 years [6]. Despite its changing epidemiology and with the incidence rising in Asia and Eastern Europe [7,8], CD is still considered a rare entity in the developing world, especially among black African patients [9,10]. We hypothesize that this disease is likely underdiagnosed due to the lack of endoscopic resources and the overlap of symptomology with schistosomiasis and tuberculosis of the bowel [4]. Review of risk factors attributable to CD remains incompletely understood despite several theories stating the interplay between infective agents, environmental, and genetic factors [11,12]. Our case cannot truly ascertain the risk factor in our patient; nevertheless, low vitamin D level is a well-studied phenomenon and associated with disease activity in CD [13]. Lifestyle factors such as stress, sleep, obesity, and exercise are poorly studied in an African context.

The presentation of CD may vary and can be insidious and nonspecific [14], and alarming symptoms as seen in our patients such as weight loss, bloody diarrhea, and other systemic manifestations should require further evaluation. We postulate that limitations of diagnostic capabilities serve as a hindrance to an established diagnosis of CD. These limitations force clinicians in low-resource settings to provide empirical care for common diseases that present in a similar fashion such as malaria and many waterborne diseases. It is likely that this patient would have benefitted from induction therapy with an anti-tumor necrosis factor (TNF) agent such as infliximab in combination with an immunomodulator such as azathioprine, which is considered first-line therapy for patients presenting with fistulizing disease. She would also need close follow-up with a dietician and surgical expertise in IBD. All of these are, sadly, hard to come by in our general setting.

## Conclusions

We presented the case as a wake-up call to the health care providers to start considering CD as a possibility in patients presenting with GI symptoms in an African context. Our case had an almost hallmark presentation of CD with both gastrointestinal and extragastrointestinal manifestations, which, unfortunately, was not picked up at her index visit. CD is considered by many as a very rare disease in Africa. The symptoms of CD may mimic many other abdominal conditions for which medical attention is required. However, it should be kept in mind as one of the causes of acute abdomen, especially in those patients who have a long history of intestinal complaints whose treatments greatly differ. A histopathologically confirmed diagnosis is necessary for the medical field especially of the emerging evidence with an increased risk of adenocarcinoma in patients with CD.
